# Transactional sex, HIV, and STIs among men who have sex with men in Ghana: an MSM bio-behavioral study

**DOI:** 10.1186/s41182-025-00821-6

**Published:** 2025-10-22

**Authors:** Chris Guure, Irene Animah Acheampong, Marian Abedua Harrision, Amos Apreku, Samuel Dery, Alhassan Yakubu, Gamji Rabiu Abu-Ba’are, Stephen Ayisi Addo, Kwasi Torpey

**Affiliations:** 1 Department of Biostatistics, School of Public Health, University of Ghana, Accra, Ghana; 2 Department of Global Health and Population, T.H. Chan School of Public Health, Harvard University, 655 Huntington Ave, Boston, USA; 3 Sakumono Specialist Hospital, Accra, Ghana; 4 Department of Population, Family and Reproductive Health, School of Public Health, University of Ghana, Accra, Ghana; 5 Department of Biostatistics, T.H. Chan School of Public Health, Harvard University, 655 Huntington Ave, Boston, USA; 6 Total Family Organisation, Accra, Ghana; 7 School of Nursing, University of Rochester, New York, USA; 8 National AIDS/STI Control Programme, Ghana Health Service, Accra, Ghana

**Keywords:** Transactional sex, MSM, HIV, Syphilis, Sexual risk

## Abstract

**Background:**

Men who have sex with men (MSM) in Ghana continue to face a disproportionately high HIV burden, with an estimated prevalence of 18.1% far exceeding the national average of 1.7%. Transactional sex (TS), defined as the exchange of sex for money, goods, or services, is a key behavioral risk factor for HIV acquisition. However, nationally representative data on its prevalence, correlates, and health consequences among MSM in Ghana remain scarce. This study aimed to identify sociodemographic and behavioral predictors of TS and assess its association with laboratory-confirmed HIV and syphilis.

**Methods:**

We conducted a cross-sectional bio-behavioral survey using respondent-driven sampling (RDS) among 3,448 MSM aged ≥ 18 years across Ghana’s ten traditional regions (August 2022–July 2023). Weighted descriptive statistics and multivariable logistic regression models were used to analyze correlates of TS and its association with HIV/syphilis. HIV and syphilis were diagnosed on-site using a rapid dual HIV–syphilis serological test, followed by confirmatory HIV testing with OraQuick and SD Bioline per Ghana’s national HIV testing guidelines.

**Results:**

Nearly half (44.8%) of participants reported engaging in TS. TS Prevalence was highest among adolescents (18–19 years: 50.8%), those with basic education (52.9%), and MSM in Greater Accra (63.9%). Older age (≥ 35 years) was associated with 63% lower odds of TS (aOR: 0.37, 95% CI 0.21–0.68, *p* = 0.001), while tertiary education was protective (aOR: 0.52, 95% CI 0.32–0.86, *p* = 0.010). TS odds increased significantly with multiple sexual partners, high alcohol use, forced sex, and poor HIV knowledge. Although TS was not independently associated with HIV (aOR: 1.16, 95% CI 0.92–1.47) or syphilis (aOR: 1.12, 95% CI 0.83–1.52), it co-occurred with other established factors.

**Conclusion:**

TS is prevalent among MSM in Ghana, driven by structural and behavioral factors such as young age (18–19), low education, urban residence, alcohol use, stigma, and sexual role. While not directly linked to HIV/syphilis risk in this study, TS serves as a significant indicator of vulnerability due to its clustering with other risks. Targeted interventions addressing these social and structural drivers, especially education and urban-focused outreach, are critical to reducing HIV transmission in this population.

## Background

The global HIV epidemic continues to disproportionately affect key populations, with men who have sex with men (MSM) representing one of the most vulnerable groups. In 2022, key populations and their sexual partners accounted for 55% of all new HIV infections worldwide, with MSM facing particularly stark disparities, showing 7.7 times higher HIV risk compared to the general population [1]. Globally, MSM remain underserved by HIV prevention programs, with limited PrEP access and persistent barriers due to stigma and discrimination [2, 3]. This heightened vulnerability arises from intersecting structural and behavioral factors, such as stigma, discrimination, social exclusion, and high-risk sexual practices, including transactional sex [4–6]. Transactional sex, defined as the exchange of sex for money, goods, or other benefits, is a key driver of HIV transmission among MSM globally. An estimated 17.4% of MSM engage in transactional sex and experience a 30% higher HIV prevalence than those who do not [7]. Systematic reviews have demonstrated wide regional variation in these patterns, underscoring the need for country-specific evidence to inform interventions[8, 9]. MSM engaged in transactional sex (MSM-TS) face multiple overlapping vulnerabilities, including structural barriers such as homelessness and limited access to healthcare; behavioral risks including unprotected sex and substance use; and socioeconomic factors that may compel engagement in transactional relationships [10]. Geographic variations in transactional sex patterns and associated HIV risk are evident across global regions [8, 9].

In the United States, where 3.7% of MSM report transactional sex, these individuals demonstrate significantly higher rates of homelessness (42%), lack of health insurance, unprotected anal sex, and illicit drug use compared to other MSM [11, 12]. These risk factors contribute to their elevated HIV/STI prevalence and reduced antiretroviral therapy utilization[12]. Similar patterns emerge in Atlanta, where 42% of gay men report transactional sex experiences, showing strong associations with gonorrhea and chlamydia co-infections[13]. Asian contexts reveal parallel concerns, with Chinese MSM-TS demonstrating sixfold higher HIV incidence rates than non-transactional MSM[14]. The African context presents complex epidemiological patterns.

In Nigeria, 38.2% of MSM engage in transactional sex, with this group showing elevated STI symptoms, though paradoxically lower HIV prevalence than non-transactional MSM. Risk factors in this population include single status, alcohol use, oral sex practices, and experiences of sexual violence[15]. Across sub-Saharan Africa more broadly, MSM-TS show 30% higher HIV prevalence and 50% greater acquisition risk[7].

Ghana reflects these disparities, with MSM HIV prevalence (17.5–18.1%) significantly higher than the national average (1.7%)[1]. A key limitation of existing work on this topic is the reliance on small, city-based samples that do not capture the full scope of transactional sex (TS) and its health implications in Ghana. Most surveys to date have drawn fewer samples from the urban cities, Accra or Kumasi, and were qualitative or behavioral in focus[16], limiting the generalizability of their findings. Moreover, to our knowledge, no nationally representative study has examined TS among Ghanaian MSM since the 2017 Ghana Men’s Study II, and no peer-reviewed analysis has linked TS to laboratory-confirmed HIV/STI outcomes in the past decade.

We aim to bridge this gap by analyzing a recent, nationally representative bio-behavioral survey to quantify the prevalence of transactional sex among men who have sex with men. We also examine biological and sociodemographic factors associated with transactional sex, as well as its relationship with HIV/STI prevalence. The findings will inform public-health programming in Ghana and the wider West African region by guiding providers on how to integrate comprehensive sexual- and reproductive-health services for people engaged in TS, shaping policy on resource allocation for STI prevention, and supporting progress toward the UNAIDS 90-90-90 cascade for HIV diagnosis, treatment, and viral suppression. Finally, this work contributes African evidence to a literature dominated by studies from other parts of the world, thereby enriching global discussions on the role of TS in HIV/STI epidemics[17].

## Methods

### Survey design and RDS recruitment

This cross-sectional survey used respondent-driven sampling (RDS). Enrollment started with purposeful seed selection. Seeds were identified during the pre-survey assessment. A detailed description of recruitment procedures, field team training, seed selection, questionnaire source, and participant identification has been published previously [18]. In brief, respondent-driven sampling (RDS) was initiated using diverse seeds at each site to enhance recruitment reach and ensure representation across subgroups within the MSM population. Between five and seven seeds per site were selected to begin the recruitment chains, each receiving three referral coupons along with guidance on peer recruitment strategies. Seeds were oriented by site supervisors on the objectives and scope of the study to foster ownership and motivation. The questionnaire used in this study was adapted from validated international bio-behavioral survey instruments developed by WHO, UNAIDS, FHI 360, CDC, and PEPFAR for populations at risk of HIV. These tools have been widely applied in multiple country contexts, ensuring comparability and methodological rigor.

### Target population

The study targeted men who have sex with men (MSM), defined as individuals assigned male at birth who reported consensual anal intercourse with another man within the past 12 months. Eligible participants were aged 18 years or older and able to communicate in English or a Ghanaian local language. All the enrolled participants met eligibility criteria and provided complete data, including laboratory-confirmed HIV and syphilis results.

### Interviewers and data collection procedures

Interviewers were primarily recruited from the MSM community with support from Civil Society Organizations (CSOs), Community-Based Organizations (CBOs), and local gatekeepers. Eligibility for field enumerator roles requires at least a Higher National Diploma (HND) qualification. Selected interviewers received structured training in research ethics, rapport building, and culturally competent interviewing techniques.

Data were collected through structured face-to-face interviews using the Research Electronic Data Capture (REDCap) system on handheld devices. Real-time data validation and daily monitoring were conducted by trained supervisors, enabling immediate resolution of inconsistencies and ensuring data quality throughout the collection process.

The study was conducted across Ghana’s ten traditional regions: Western, Central, Eastern, Greater Accra, Volta, Ashanti, Brong Ahafo, Northern, Upper East, and Upper West. Ghana is situated in West Africa, bordered by Côte d'Ivoire, Burkina Faso, and Togo. As of 2021, the country has an estimated population of 32.8 million.

### Sample size calculation

The sample size was estimated based on the assumption of a 48.2% baseline prevalence of consistent condom use among MSM, as reported by the Ghana AIDS Commission (2017) [19], to detect a 15% absolute difference. Using a significance level (α) of 0.05 and 80% statistical power, and accounting for a design effect of 1.5 and a projected 10% nonresponse rate, the required sample size was calculated to be approximately 342 participants per site. Considering the ten traditional regions (strata), this yielded a total target sample of 3,420 MSM nationwide.

### Study variables

#### Outcome variables

The primary outcome variable was engagement in transactional sex, defined as the exchange of sex for money, goods, or services. This was assessed through the question: “Have you ever received money, goods, or favors in exchange for sex?” with responses categorized as “yes” or “no”. For the secondary objective, two binary outcomes were considered: laboratory-confirmed HIV status and syphilis status, both determined through rapid dual HIV–syphilis serological tests, followed by confirmatory HIV testing with OraQuick and SD Bioline per Ghana’s national HIV testing guidelines.

#### Predictor variables

The study included a range of sociodemographic, behavioral, and psychosocial predictors. Sociodemographic variables included age group, level of education, marital status, region of residence, and monthly income. Behavioral variables included the number of sexual partners in the past 6 months, alcohol consumption, and knowledge of HIV testing sites. Psychosocial variables captured included attraction to the same or opposite sex, prior experience of forced sex, stigma experience, and awareness of pre-exposure prophylaxis (PrEP). HIV knowledge was assessed using a composite indicator based on responses to multiple questions covering modes of transmission and prevention. All variables were treated as categorical, and where relevant, nonresponse or “don’t know” categories were retained for analysis.

### Statistical analysis

Both descriptive and inferential statistical methods were employed in this study. Weighted frequencies and percentages were used to describe the prevalence of transactional sex, HIV, and syphilis across sociodemographic and behavioral subgroups. The prevalence estimates and their corresponding 95% confidence intervals were computed for key variables.

Bivariate logistic regression analyses were conducted to examine the association between transactional sex and various sociodemographic, behavioral, and psychosocial predictors. Separate logistic regression models were also fitted to assess the association between transactional sex and laboratory-confirmed HIV and syphilis outcomes. Variables that were statistically significant at the *p* < 0.05 level in bivariate models were considered for inclusion in multivariable models. In addition, key variables informed by existing literature, such as age, education level, sexual partnership patterns, HIV testing history, and experience of stigma or forced sex, were retained in multivariable models regardless of their statistical significance to ensure theoretical robustness.

All analyses were weighted to account for the respondent-driven sampling (RDS) design. Individual participant weights were derived using RDS Analyst software, based on reported network size and recruitment patterns. Statistical significance was set at an alpha level of 0.05, and adjusted odds ratios (aORs) with 95% confidence intervals (CIs) were reported. All analyses were performed using Stata MP version 18.5 (StataCorp, College Station, TX, USA).

### Determination of sampling weights

Sampling weights were computed using the RDS Analyst software to adjust for the non-random nature of respondent-driven sampling (RDS). For each region, data were uploaded into the software using key recruitment and network information, including participant and coupon identifiers, individual network sizes, and estimated MSM population sizes.

Network size was assessed using a sequence of questions designed to progressively narrow a participant’s social reach within the MSM community. Participants were asked: (1) “How many MSM do you currently know who are part of the community?”; (2) “Of these, how many live, work, or socialize in this area?”; (3) “How many of those are aged 18 years and older?”; (4) “How many have you seen in the last 30 days?”; (5) “How many have you spoken to in the last two weeks?”; and (6) “Of those you spoke to in the last two weeks, how many would you feel comfortable inviting to participate in this survey?” The response to the final question was used as the participant’s reported network size for weighting purposes.

These individualized weights were then incorporated into all analyses to ensure that the results reflected population-level estimates across Ghana’s MSM community.

## Results

### Characteristics of study participants

Among 3,448 participants included in the analysis, nearly half (48.1%) were aged 20–24 years, while 4.5% were aged 35 years and above. More than two-thirds (73.3%) had attained at least senior high school education, with the majority having senior high school education (53.3%). The majority (92.8%) were single and never married. Only 16.1% reported currently living with a partner. Geographically, the sample was broadly distributed, with the highest representation from Greater Accra (18.9%), followed by Western (15.7%) and Ashanti (13.2%) regions, while the Upper East (2.2%) and Upper West (2.4%) regions had the fewest participants.

For sexual orientation, 34.2% of participants reported being mostly attracted to males, and 30.7% exclusively to males. One-quarter (25.8%) had never had vaginal sex, and a similar proportion (24.5%) reported never having had anal sex with a woman. Over half (56.1%) reported never consuming alcohol, and 45.2% had contact with a peer educator in the past 12 months.

Most participants (68.4%) reported ever testing for HIV, while only 14.3% had ever experienced stigma. Less than half (46.0%) demonstrated comprehensive knowledge of HIV. Forced sex was reported by 11.2% of respondents, with 3.4% experiencing it once. Income distribution showed that 38.9% earned less than GHS 500 per month, while only 11.5% earned GHS 2000 or more.

Majority (91.7%) reported having had a regular sexual partner in the past six months, and 62.7% had also had a non-regular partner. A little over half (52.2%) had heard of PrEP, but 45.0% were willing to take it. Most respondents (86.4%) knew where to test for HIV.

About two in five participants (41.8%) reported using a condom during their last anal sex, while 30.2% did not, and 28.1% did not provide a response. Regarding anal sex type, over half (51.2%) reported engaging exclusively in insertive sex, 15.5% exclusively receptive, and 31.1% both insertive and receptive (Table 1).

**Table 1:** Sociodemographic characteristics of study participants (N = 3448)

Variable	Frequency	Percentage
*Age group*		
18–19	376	10.91
20–24	1659	48.14
25–34	1255	36.4
≥ 35	154	4.47
Nonresponse	2	0.07
*Education*		
Basic (none/primary)	154	4.46
Junior high school	766	22.23
Senior high school	1834	53.21
Tertiary	691	20.05
Nonresponse	2	0.04
*Marital status*		
Single, never married	3199	92.8
Married/cohabiting	202	5.86
Separated/divorced/widowed	39	1.12
Nonresponse	8	0.23
*Currently living with a partner*		
No	2888	83.77
Yes	556	16.12
Nonresponse	4	0.11
*Region*		
Greater Accra	650	18.87
Ashanti	454	13.18
Volta	329	9.56
Western	541	15.68
Eastern	400	11.61
Central	322	9.33
Brong Ahafo	399	11.56
Northern	193	5.61
Upper East	74	2.16
Upper West	84	2.43
*Sex attracted to most*		
Only male	1057	30.67
Mostly male	1178	34.18
Equally male and female	966	28.03
Mostly female	231	6.7
Nonresponse	14	0.41
*History of vaginal sex*		
None	887	25.75
1 Woman	715	20.75
2 women	403	11.7
3 + Women	578	16.75
Never had anal sex with a woman	846	24.53
Nonresponse	18	0.52
*Alcohol consumption*		
Never	1935	56.14
Moderate	1190	34.53
High	314	9.12
Nonresponse	7	0.21
*Contact with peer educator*		
No	1873	54.35
Yes	1557	45.17
Nonresponse	17	0.49
*Ever tested for HIV*		
Yes	2359	68.44
No	1072	31.09
Nonresponse	16	0.47
*Experienced stigma*		
Yes	493	14.31
No	2930	85
Nonresponse	24	0.69
*Comprehensive knowledge on HIV*		
Yes	1585	45.99
No	1862	54.01
*Forced sex*		
Never	3,062	88.84
Once	118	3.42
A few times	55	1.6
Often	10	0.28
Does not apply because no one knows	195	5.65
Nonresponse	8	0.22
*Income*		
< Ghc500.00	1344	38.99
Ghc500-999.00	949	27.52
Ghc1000-1999.00	708	20.53
Ghc2000.00 +	396	11.5
Nonresponse	50	1.46
*Condom use during last anal sex*		
No condom used	1039	30.15
Condom used	1441	41.79
Nonresponse	967	28.06
*Anal sex type ever experienced*		
Insertive only	1774	51.23
Receptive only	536	15.48
Both insertive and receptive	1112	31.11
Nonresponse	41	1.18
*Had regular sex partner in the last 6 months*		
No	283	8.21
Yes	3161	91.69
Nonresponse	3	0.1
*Had a non-regular sex partner in the last 6 months*		
No	1273	36.93
Yes	2160	62.66
Nonresponse	14	0.41
*Ever heard of PrEP*		
Yes	1800	52.22
No	1636	47.47
Nonresponse	11	0.32
*Willingness to take PrEP*		
Yes	1550	44.96
No	188	5.45
Nonresponse	1709	49.59
*Know where to test for HIV*		
Yes	2978	86.4
No	451	13.07
Nonresponse	18	0.53

### Prevalence of transactional sex among MSM in Ghana

**Fig. 1:**
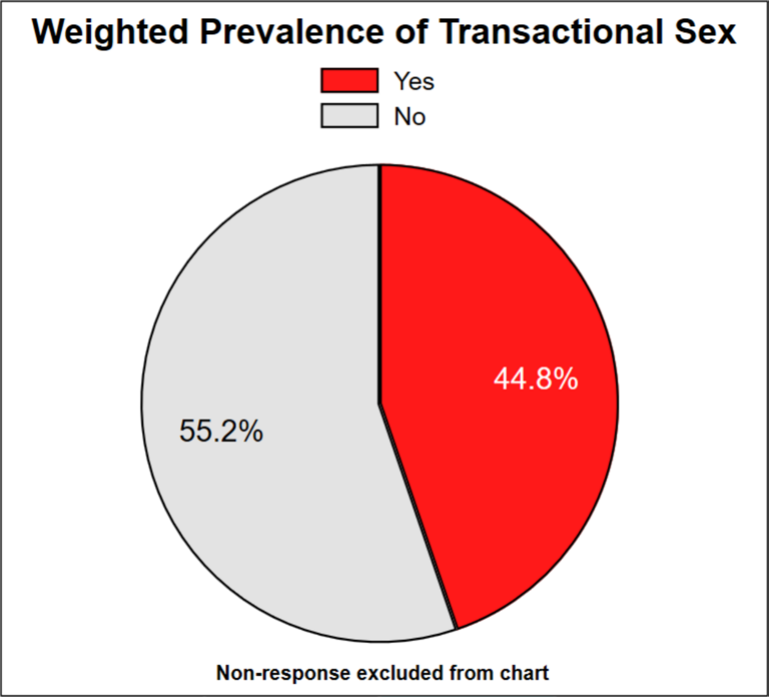
Prevalence of transactional sex among MSM in Ghana

The weighted prevalence of transactional sex among MSM was 44.8%. This suggests that nearly one in two participants reported having ever received money or goods in exchange for sex (Fig. 1).

### Sexual practices and condom use among MSM engaged in transactional sex

Among participants reporting transactional sex, one-third (32.9%) had one paying sexual partner in the past six months, while 31.2% reported three or more. The majority engaged in insertive anal sex with their last paying partner (54.6%), followed by receptive (32.5%) and both roles (12.2%). Regarding condom use with paying partners in the last year, about half (51.3%) reported sometimes using condoms, 33.6% always, and 13.4% never (Table 2).

**Table 2:** Characteristics of sexual practices and condom use among MSM who engaged in transactional sex in the past 6 months

Characteristics	*N* (%)
*Number of paying sexual partners in the last 6 months*	
None	186 (12.3)
1 person	497 (32.9)
2 people	356 (23.5)
3 + people	472 (31.2)
Nonresponse	1 (0.1)
*Type of anal sex with last paying partner*	
Receptive	492 (32.5)
Insertive	826 (54.6)
Both	185 (12.2)
Nonresponse	9 (0.6)
*Frequency of condom use with paying partner in the last 12 months*	
Always	508 (33.6)
Sometimes	775 (51.3)
Never	202 (13.4)
Nonresponse	27 (1.8)

### Factors associated with transactional sex among MSM in Ghana

From the bivariate analysis, transactional sex was more prevalent among MSM aged 18–19 years (50.8%), those with basic education (52.9%), and those residing in Greater Accra (63.9%). Higher rates were also observed among MSM who were mostly attracted to females (49.6%), had four or more sexual partners in the past 6 months (59.7%), consumed alcohol at high levels (55.7%), and had experienced forced sex (66.9–80.0%). Participants who had not heard of PrEP (41.8%) were significantly less likely to report transactional sex compared to those who had heard of PrEP. Participants who did not experience stigma (42.4%) were significantly less likely to report transactional sex compared to those who had experienced stigma. Transactional sex was least prevalent among participants aged 35 years and above (29.7%), tertiary-educated (34.0%), and participants from Upper East (16.0%) and Upper West (19.0%) regions.

From the multivariable logistic regression model, MSM aged 35 years and above had 62% lower odds of engaging in transactional sex compared to adolescents aged 18–19 (aOR: 0.38; 95% CI 0.21–0.69; *p* = 0.001), while those aged 25–34 years had 34% lower odds also compared to the 18–19 years old (aOR: 0.66; 95% CI 0.47–0.95; *p* = 0.023). Participants with tertiary education also had reduced odds compared to those with basic education (aOR: 0.54; 95% CI 0.33–0.89; *p* = 0.016). MSM who were separated/divorced/widowed also had lower odds of transactional sex (aOR: 0.40; 95% CI 0.15–1.07; *p* = 0.008).

Condom use during last anal sex was not significantly associated with transactional sex (aOR: 1.00; 95% CI 0.78–1.27; *p* = 0.970). However, MSM who reported receptive-only (aOR: 2.15; 95% CI 1.58–2.93; *p* < 0.001) or both insertive and receptive anal sex (aOR: 1.52; 95% CI 1.19–1.93; *p* = 0.001) had significantly higher odds of engaging in transactional sex compared to those who were exclusively insertive.

Regionally, MSM from all the other regions had significantly lower odds compared to the Greater Accra region, with Volta (aOR: 0.27; 95% CI 0.18–0.42), Central (aOR: 0.23; 95% CI 0.15–0.36), Northern (aOR: 0.22; 95% CI 0.14–0.35), and Upper East (aOR: 0.11; 95% CI 0.06–0.22) having the least odds of engaging in transactional sex.

A higher number of sexual partners was strongly associated with transactional sex: MSM with 4 + partners had nearly twice the odds compared to those with 1 partner (aOR: 1.91; 95% CI 1.36–2.69; *p* < 0.001). Other significant correlates included high alcohol consumption (aOR: 1.61; 95% CI 1.13–2.30; *p* = 0.007) and ever experiencing forced sex (aOR: 1.75; 95% CI 1.04–2.95; *p* = 0.036) according to this sample data (Table 3).

**Table 3:** Factors associated with transactional sex among MSM in Ghana

	Transactional sex		Binary logistic regression model of TS among MSM			
			Unadjusted model		Adjusted model	
Characteristics	n/N (%)	*p*-value	cOR [95% CI]	*p*-value	aOR [95% CI]	*p*-value
*Age group*		0.003				
18–19	192 /378 (50.8)		1.00 [reference]		1.00 [reference]	
20–24	775 /1667 (46.5)		0.84 [0.63, 1.12]	0.240	0.87 [0.63, 1.20]	0.381
25–34	534 /1260 (42.4)		0.71 [0.53, 0.96]	0.025	0.66 [0.47, 0.95]	0.023
≥ 35	46 /155 (29.7)		0.41 [0.25, 0.68]	< 0.001	0.38 [0.21, 0.69]	0.001
Nonresponse	2 /2 (100.0)		–	–	–	–
*Education*		< 0.001				
Basic (none/primary)	82 /155 (52.9)		1.00 [reference]		1.00 [reference]	
Junior high school	380 /770 (49.4)		0.86 [0.55, 1.33]	0.490	1.02 [0.63, 1.63]	0.947
Senior high school	850 /1842 (46.1)		0.75 [0.50, 1.13]	0.174	0.82 [0.53, 1.28]	0.391
Tertiary	236 /694 (34.0)		0.45 [0.29, 0.70]	< 0.001	0.54 [0.33, 0.89]	0.016
Nonresponse	2 /2 (100.0)		–	–	–	–
*Marital status*		0.006				
Single, never married	1458 /3213 (45.4)		1.00 [reference]		1.00 [reference]	
Married/cohabiting	78 /203 (38.4)		0.76 [0.52, 1.11]	0.154	0.76 [0.50, 1.16]	0.068
Separated/divorced/widowed	8 /39 (20.5)		0.30 [0.13, 0.73]	0.008	0.40 [0.15, 1.07]	0.008
Nonresponse	6 /8 (75.0)		–	–	–	–
*Currently living with a partner*		0.900		1.940	–	–
No	1293 /2900 (44.6)		1.00 [reference]			
Yes	255 /558 (45.7)		1.05 [0.83, 1.33]	0.704	–	–
Nonresponse	2 /4 (50.0)		–	–	–	–
*Region*		< 0.001				
Greater Accra	417 /653 (63.9)		1.00 [reference]		1.00 [reference]	
Ashanti	251 /456 (55.0)		0.69 [0.52, 0.93]	< 0.001	0.73 [0.50, 1.05]	0.090
Volta	101 /331 (30.5)		0.25 [0.17, 0.36]	< 0.001	0.27 [0.18, 0.42]	< 0.001
Western	247 /543 (45.5)		0.47 [0.34, 0.66]	< 0.001	0.50 [0.34, 0.75]	< 0.001
Eastern	207 /402 (51.5)		0.60 [0.42, 0.84]	< 0.001	0.53 [0.34, 0.84]	0.007
Central	100 /323 (31.0)		0.25 [0.18, 0.36]	< 0.001	0.23 [0.15, 0.36]	< 0.001
Brong Ahafo	149 /400 (37.3)		0.33 [0.24, 0.48]	< 0.001	0.31 [0.20, 0.47]	< 0.001
Northern	51 /194 (26.3)		0.20 [0.13, 0.30]	< 0.001	0.22 [0.14, 0.35]	< 0.001
Upper East	12 /75 (16.0)		0.10 [0.06, 0.17]	< 0.001	0.11 [0.06, 0.22]	< 0.001
Upper West	16 /84 (19.0)		0.13 [0.08, 0.21]	< 0.001	0.13 [0.06, 0.22]	< 0.001
*Sex attracted to most*		0.014				
Only male	514 /1062 (48.4)		1.00 [reference]		1.00 [reference]	
Mostly male	535 /1184 (45.2)		0.88 [0.71, 1.10]	< 0.001	0.82 [0.62, 1.08]	0.155
Equally male and female	379 /971 (39.0)		0.68 [0.54, 0.86]	< 0.001	0.74 [0.54, 1.02]	0.064
Mostly female	115 /232 (49.6)		1.05 [0.70, 1.58]	0.220	0.94 [0.56, 1.57]	0.801
Nonresponse	7 /14 (50.0)		–	–	–	–
*History of vaginal sex*		< 0.001				
None	390 /891 (43.8)		1.00 [reference]		1.00 [reference]	
1 Woman	291 /718 (40.5)		0.88 [0.67, 1.15]	< 0.001	1.13 [0.82, 1.54]	0.459
2 women	172 /405 (42.5)		0.95 [0.70, 1.31]	< 0.001	1.09 [0.76, 1.55]	0.647
3 + Women	319 /580 (55.0)		1.57 [1.18, 2.09]	3.140	1.39 [0.98, 1.97]	0.062
Never had anal sex with a woman	374 /849 (44.1)		1.01 [0.79, 1.30]	0.110	0.63 [0.46, 0.86]	0.004
Nonresponse	4 /18 (22.2)		–	–	–	–
*Sexual partners*		< 0.001				
1 Partners	224 /788 (28.4)		1.00 [reference]		1.00 [reference]	
2 Partners	319 /850 (37.5)		1.51 [1.16, 1.98]	3.010	1.18 [0.84, 1.63]	0.339
3 Partners	257 /568 (45.2)		2.08 [1.55, 2.79]	4.880	1.37 [0.95, 1.97]	0.090
4 + Partners	749 /1254 (59.7)		3.74 [2.91, 4.81]	10.290	1.91 [1.36, 2.69]	< 0.001
Nonresponse	1 /2 (50.0)		–	–	–	–
*Alcohol consumption*		0.003				
Never	822 /1944 (42.3)		1.00 [reference]		1.00 [reference]	
Moderate	550 /1195 (46.0)		1.16 [0.96, 1.41]	1.520	1.11 [0.89, 1.40]	0.361
High	176 /316 (55.7)		1.71 [1.25, 2.33]	3.340	1.61 [1.13, 2.30]	0.007
Nonresponse	2 /7 (28.6)		–	–	–	–
*Contact with peer educator*		0.267				
No	857 /1882 (45.5)		1.00 [reference]		1.00 [reference]	
Yes	688 /1564 (44.0)		0.94 [0.79, 1.12]	< 0.001	0.79 [0.57, 1.09]	0.150
Nonresponse	4 /17 (23.5)		–	–	–	–
*Ever tested for HIV*		0.050				
Yes	1093 /2370 (46.1)		1.00 [reference]			
No	452 /1076 (42.0)		0.85 [0.70, 1.02]	< 0.001	–	–
Nonresponse	4 /16 (25.0)		–	–	–	–
*Experienced stigma*		< 0.001				
Yes	295 /496 (59.5)		1.00 [reference]		1.00 [reference]	
No	1248 /2943 (42.4)		0.50 [0.39, 0.64]	< 0.001	0.71 [0.53, 0.95]	0.023
Nonresponse	7 /24 (29.2)		–	–	–	–
*Comprehensive knowledge on HIV*		0.020				
Yes	668 /1592 (42.0)		1.00 [reference]		1.00 [reference]	
No	881 /1870 (47.1)		1.23 [1.03, 1.47]	2.330	0.97 [0.79, 1.20]	0.799
*Forced sex*		< 0.001				
Never	1355 /3076 (44.1)		1.00 [reference]		1.00 [reference]	
Once	79 /118 (66.9)		2.56 [1.54, 4.26]	3.640	1.75 [1.04, 2.95]	0.036
A few times	34 /55 (61.8)		2.08 [0.93, 4.67]	1.770	1.37 [0.48, 3.95]	0.557
Often	8 /10 (80.0)		4.86 [1.19, 19.81]	2.210	3.39 [0.84, 13.66]	0.087
Does not apply because no one knows	72 /196 (36.7)		0.74 [0.48, 1.12]	< 0.001	0.81 [0.50, 1.31]	0.383
Nonresponse	1 /8 (12.5)		–	–	–	–
*Income*		0.357				
< Ghc500.00	586 /1350 (43.4)		1.00 [reference]		1.00 [reference]	
Ghc500-999.00	429 /953 (45.0)		1.07 [0.85, 1.33]	0.570	1.01 [0.78, 1.30]	0.944
Ghc1000-1999.00	347 /711 (48.8)		1.25 [0.98, 1.59]	1.770	1.13 [0.86, 1.52]	0.389
Ghc2000.00 +	168 /398 (42.2)		0.95 [0.71, 1.27]	< 0.001	0.98 [0.71, 1.37]	0.916
Nonresponse	21 /51 (41.2)		–	–	–	–
*Condom use during last anal sex*		0.010				
No condom used	466 /1044 (44.6)		1.00 [reference]		1.00 [reference]	
Condom used	600 /1447 (41.5)		0.88 [0.71, 1.09]	< 0.001	1.00 [0.78, 1.27]	0.970
Nonresponse	483 /972 (49.7)		1.23 [0.98, 1.55]	1.750	0.97 [0.73, 1.29]	0.819
*Anal sex type ever experienced*		< 0.001				
Insertive only	694 /1774 (39.1)		1.00 [reference]		1.00 [reference]	
Receptive only	278 /535 (52.0)		1.68 [1.30, 2.17]	4.000	2.15 [1.58, 2.93]	< 0.001
Both insertive and receptive	568 /1112 (51.1)		1.63 [1.33, 1.99]	4.760	1.52 [1.19, 1.93]	0.001
Nonresponse	9 /41 (22.0)		0.43 [0.14, 1.30]	< 0.001	0.78 [0.15, 3.99]	0.767
*Had regular sex partner in the last 6 months*		0.476				
No	119 /284 (41.9)		1.00 [reference]			
Yes	1430 /3175 (45.0)		1.14 [0.82, 1.58]	0.780	–	–
Nonresponse	1 /3 (33.3)		–	–	–	–
*Had a non-regular sex partner in the last 6 months*		< 0.001				
No	373 /1279 (29.2)		1.00 [reference]			
Yes	1172 /2169 (54.0)		2.85 [2.35, 3.45]	10.690	–	–
Nonresponse	5 /14 (35.7)		–	–	–	–
*Ever heard of PrEP*		< 0.001				
Yes	863 /1808 (47.7)		1.00 [reference]		1.00 [reference]	
No	686 /1643 (41.8)		0.78 [0.66, 0.94]	< 0.001	0.47 [0.25, 0.89]	0.019
Nonresponse	0 /11 (0.0)		–	–	–	–
*Willingness to take PrEP*		0.013				
Yes	724 /1557 (46.5)		1.00 [reference]		1.00 [reference]	
No	104 /189 (55.0)		1.41 [0.94, 2.13]	1.660	1.13 [0.70, 1.81]	0.619
Nonresponse	722 /1717 (42.1)		–	–	–	–
*Know where to test for HIV*		0.192				
Yes	1344 /2991 (44.9)		1.00 [reference]			
No	201 /453 (44.4)		0.98 [0.76, 1.27]	< 0.001	–	–
Nonresponse	4 /19 (21.1)		–	–	–	–

n/N (%): frequency TS /total frequency (percentage). COR: crude odds ratio. aOR: adjusted odds ratio. CI confidence interval

### Association of transactional sex and other correlates with HIV and syphilis among MSM in Ghana

**Fig. 2:**
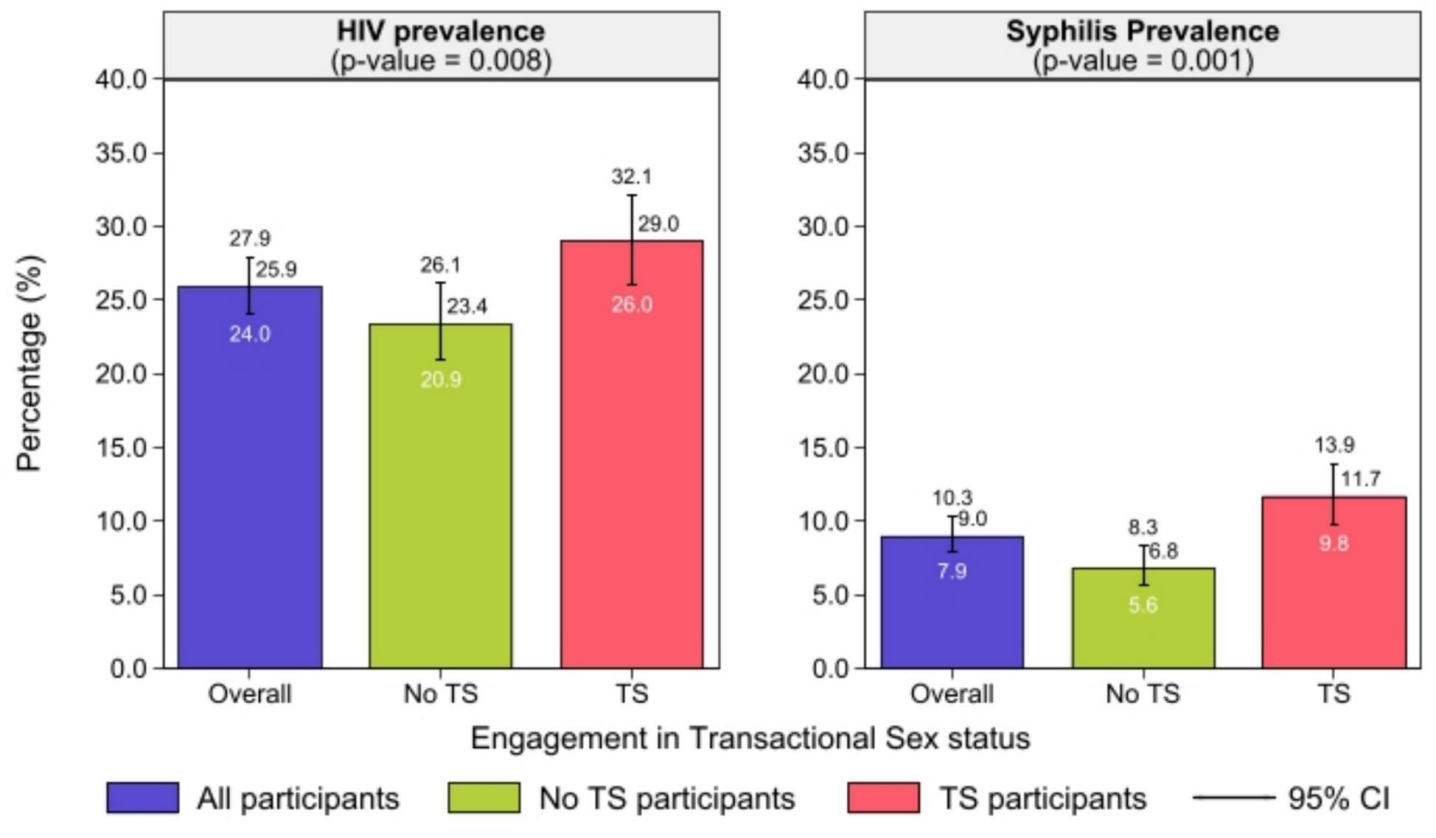
Bivariate association between transactional sex and HIV and syphilis prevalence among MSM in Ghana

From the bivariate analysis, participants who engaged in TS have a high prevalence of HIV (29%) and syphilis (11.7%) compared to those who do not engage in transactional sex (23.4% for HIV; 6.8% for syphilis) (Fig. 2).

From the multivariate logistic regression, transactional sex was not significantly associated with HIV (aOR: 1.02; 95% CI 0.79–1.32; *p* = 0.869) or syphilis infection (aOR: 1.05; 95% CI 0.74–1.48; *p* = 0.800). MSM aged ≥ 35 years had markedly higher odds of being HIV-positive compared to those aged 18–19 (aOR: 7.65; 95% CI 3.49–16.77; *p* < 0.001). Not living with a partner was also associated with increased odds of HIV (aOR: 1.58; 95% CI 1.10–2.26; *p* = 0.012). For sexual behavior variables, MSM who reported receptive-only anal sex (aOR: 6.43; 95% CI 4.40–9.40; *p* < 0.001) or both insertive and receptive roles (aOR: 7.39; 95% CI 5.50–9.92; *p* < 0.001) had substantially higher odds of being HIV-positive compared to those who were exclusively insertive. Lack of HIV testing was strongly associated with HIV infection (aOR: 0.36; 95% CI 0.26–0.51; *p* < 0.001). PrEP awareness was protective against HIV (aOR: 0.26; 95% CI 0.13–0.52; *p* < 0.001), while willingness to take PrEP was associated with higher odds of HIV infection (aOR: 2.37; 95% CI 1.45–3.89; *p* = 0.001).

For syphilis, significantly lower odds were observed among MSM from Western, Volta, Brong Ahafo, Northern, Upper East, and Upper West compared to Greater Accra. Sexual attraction mostly to males (aOR: 0.59; 95% CI 0.39–0.89; *p* = 0.011) or equally to males and females (aOR: 0.53; 95% CI 0.32–0.90; *p* = 0.015) was protective. Receptive-only (aOR: 2.37; 95% CI 1.40–3.99; *p* = 0.001) and dual-role anal sex (aOR: 3.67; 95% CI 2.43–5.54; *p* < 0.001) were associated with higher odds of syphilis (Table 4).

**Table 4:** Association of transactional sex and other correlates with HIV and syphilis among MSM in Ghana

	Binary logistic regression model			
	Adjusted model (HIV)		Adjusted model (syphilis)	
Characteristics	aOR [95% CI]	P-value	aOR [95% CI]	P-value
*Transactional sex*				
No	1.00 [reference]		1.00 [reference]	
Yes	1.02 [0.79, 1.32]	0.869	1.05 [0.74, 1.48]	0.800
*Age group*				
18–19	1.00 [reference]		1.00 [reference]	
20–24	1.45 [0.91, 2.31]	0.120	0.76 [0.43, 1.36]	0.359
25–34	2.41 [1.49, 3.90]	< 0.001	0.99 [0.54, 1.81]	0.972
≥ 35	7.65 [3.49, 16.77]	< 0.001	0.86 [0.34, 2.16]	0.747
Nonresponse	1.00 [0.00, 0.00]		1.00 [0.00, 0.00]	
*Highest education*				
Basic (none/primary)	1.00 [reference]		1.00 [reference]	
Junior high school	0.79 [0.43, 1.45]	0.443	0.85 [0.43, 1.67]	0.627
Senior high school	0.97 [0.55, 1.73]	0.923	0.75 [0.40, 1.40]	0.359
Tertiary	1.05 [0.57, 1.95]	0.875	0.77 [0.39, 1.53]	0.460
Nonresponse	1.00 [0.00, 0.00]		1.00 [0.00, 0.00]	
*Marital status*				
Single, never married	1.00 [reference]		1.00 [reference]	
Married/cohabiting	0.77 [0.45, 1.34]	0.357	0.67 [0.33, 1.33]	0.252
Separated/divorced	0.65 [0.21, 1.95]	0.440	1.01 [0.23, 4.39]	0.993
Widowed	1.00 [0.00, 0.00]		18.29 [3.52, 95.13]	0.001
Nonresponse	0.30 [0.08, 1.19]	0.088	2.97 [0.60, 14.71]	0.183
*Currently living with a partner*				
Yes	1.00 [reference]		1.00 [reference]	
No	1.58 [1.10, 2.26]	0.012	1.26 [0.80, 1.99]	0.314
Nonresponse	1.00 [0.00, 0.00]		1.00 [0.00, 0.00]	
*Region*				
Greater Accra	1.00 [reference]		1.00 [reference]	
Ashanti	0.92 [0.63, 1.34]	0.676	0.43 [0.27, 0.68]	< 0.001
Volta	0.78 [0.50, 1.22]	0.275	0.42 [0.23, 0.76]	0.005
Western	0.59 [0.39, 0.91]	0.017	0.49 [0.28, 0.87]	0.015
Eastern	1.55 [0.97, 2.49]	0.069	0.72 [0.44, 1.18]	0.188
Central	0.53 [0.32, 0.90]	0.018	0.75 [0.44, 1.29]	0.298
Brong Ahafo	0.50 [0.31, 0.80]	0.004	0.09 [0.04, 0.21]	< 0.001
Northern	0.29 [0.16, 0.53]	< 0.001	0.07 [0.02, 0.20]	< 0.001
Upper East	1.00 [0.00, 0.00]		1.00 [0.00, 0.00]	
Upper West	0.14 [0.05, 0.38]	< 0.001	0.04 [0.01, 0.18]	< 0.001
*Sex attracted to most*				
Only male	1.00 [reference]		1.00 [reference]	
Mostly male	0.95 [0.69, 1.30]	0.746	0.59 [0.39, 0.89]	0.011
Equally male and female	0.94 [0.63, 1.38]	0.740	0.53 [0.32, 0.89]	0.015
Mostly female	0.73 [0.34, 1.55]	0.407	0.23 [0.09, 0.61]	0.003
Nonresponse	0.65 [0.07, 5.65]	0.692	0.85 [0.05, 14.43]	0.912
*History of vaginal sex with women in the past 6 months*				
None	1.00 [reference]		1.00 [reference]	
1 woman	0.56 [0.38, 0.82]	0.003	0.67 [0.41, 1.08]	0.100
2 women	0.74 [0.46, 1.18]	0.205	1.19 [0.65, 2.17]	0.569
3 + women	0.59 [0.38, 0.91]	0.018	0.74 [0.43, 1.28]	0.288
Had anal sex with woman	0.88 [0.63, 1.24]	0.475	0.70 [0.46, 1.09]	0.117
Nonresponse	0.68 [0.16, 2.93]	0.600	3.12 [0.60, 16.20]	0.175
*Anal sex type ever experienced*				
Insertive only	1.00 [reference]		1.00 [reference]	
Receptive only	6.43 [4.40, 9.40]	< 0.001	2.37 [1.40, 3.99]	0.001
Both insertive and receptive	7.39 [5.50, 9.92]	< 0.001	3.67 [2.43, 5.54]	< 0.001
Nonresponse	9.52 [2.48, 36.54]	0.001	0.52 [0.05, 5.24]	0.581
*Sexual partners*				
1 Partners	1.00 [reference]		1.00 [reference]	
2 Partners	0.73 [0.51, 1.04]	0.084	0.81 [0.49, 1.33]	0.409
3 Partners	0.59 [0.40, 0.88]	0.009	1.04 [0.61, 1.75]	0.892
4 + Partners	0.91 [0.66, 1.26]	0.562	1.59 [1.03, 2.46]	0.037
Nonresponse	0.68 [0.06, 7.33]	0.749	3.56 [0.21, 59.36]	0.377
*Ever tested for HIV*				
Yes	1.00 [reference]		1.00 [reference]	
No	0.36 [0.26, 0.51]	< 0.001	0.43 [0.26, 0.70]	0.001
Nonresponse	0.11 [0.01, 1.41]	0.090	1.18 [0.10, 13.94]	0.893
*Experienced stigma*				
Yes	1.00 [reference]		1.00 [reference]	
No	1.05 [0.76, 1.45]	0.779	0.77 [0.53, 1.13]	0.180
Nonresponse	0.44 [0.10, 1.88]	0.270	1.00 [0.00, 0.00]	
*Ever heard of PrEP*				
Yes	1.00 [reference]		1.00 [reference]	
No	0.26 [0.13, 0.52]	< 0.001	0.52 [0.21, 1.30]	0.164
Nonresponse	10.25 [1.54, 68.38]	0.016	1.00 [0.00, 0.00]	
*Will take PrEP to prevent HIV*				
Yes	1.00 [reference]		1.00 [reference]	
No	2.37 [1.45, 3.89]	0.001	1.10 [0.65, 1.88]	0.722
Nonresponse	2.72 [1.40, 5.28]	0.003	1.31 [0.54, 3.19]	0.550

## Discussion

This study provides insights into the factors driving engagement in transactional sex among MSM in Ghana. In addition to identifying predictors of transactional sex, we also examined its association with HIV and syphilis. Although TS was not independently associated with HIV or syphilis, it clustered with multiple behavioral and structural vulnerabilities that heighten overall risk. The findings underscore the need for targeted interventions that address the unique contexts in which TS occurs.

Transactional sex was most prevalent among adolescents aged 18–19 years, with odds decreasing significantly with age. This trend is consistent with global literature suggesting that younger MSM are more likely to engage in TS due to economic vulnerability, limited employment opportunities, and social exclusion [20–23]. In Ghana, the high youth unemployment rate and lack of income-generating alternatives may push adolescents into transactional arrangements as a form of survival.

Education emerged as a significant protective factor. MSM with tertiary education had significantly lower odds of engaging in transactional sex compared to those with lower educational attainment. This finding is consistent with several studies showing that education enhances employability, economic independence, and health literacy—factors that reduce reliance on transactional partnerships [12, 20, 22–24]. Educated individuals may also have increased access to health information and support networks, reducing their reliance on transactional partnerships.

MSM who were separated, divorced, or widowed were also less likely to engage in transactional sex. This finding contrasts with some previous studies that have shown higher odds of engaging in transactional sex among individuals who are single or not in a current relationship, where the absence of a committed partner is often associated with increased sexual risk-taking [23].

This may reflect a combination of age, emotional fatigue, and social withdrawal following the dissolution of a prior relationship. In the Ghanaian context, individuals who have experienced the emotional toll of long-term relationships may become more risk-averse, potentially distancing themselves from casual or transactional sexual partnerships. The trauma of a previous union might contribute to lower sexual activity overall, including engagement in economically motivated encounters.

Geographic disparities were also evident. MSM residing in the Volta, Northern, and Upper East regions had significantly lower odds of engaging in transactional sex compared to those in Greater Accra. As Ghana’s capital and most urbanized region, Greater Accra presents unique structural drivers of transactional sex, including a high cost of living, widespread youth underemployment, and housing instability [20, 25, 26]. The city also attracts internal migrants seeking better opportunities, many of whom may face economic precarity and lack social support networks, thereby increasing their vulnerability to exchanging sex for shelter or basic necessities. Urban environments such as Greater Accra also offer greater anonymity and lower risk of social sanction, which can facilitate the normalization or concealment of transactional sex behaviors.

The findings echo broader patterns observed globally, where transactional sex is often more prevalent in urban centers due to concentrated economic pressures and greater exposure to commercialized sexual networks[7]. While the Greater Accra Region has been the focal point for HIV prevention programming in Ghana, this study highlights the need to consider how social and structural inequalities within urban settings may paradoxically increase vulnerability even in regions with high programmatic coverage. Economic strain, social isolation, and migratory instability may dilute the protective effects of health interventions unless they are explicitly designed to address these contextual realities.

In this study, income level was not found to be associated with transactional sex, which contrasts with several prior studies conducted globally. While this finding may reflect context-specific dynamics, it diverges from a well-established trend observed in research from Africa, North America, and Europe, where lower income has consistently been linked to a higher likelihood of engaging in transactional sex among MSM [20, 21, 27]. In those contexts, financial insecurity often compels individuals to exchange sex for money, shelter, or material support. The deviation observed in this study may suggest that the motivations for transactional sex are multifaceted and not only shaped by absolute income but also by local social networks, housing instability, access to employment, and prevailing norms within the MSM community. Further investigation is needed to unpack the nuanced role of income and economic opportunity in shaping transactional sex behaviors in this setting.

MSM who were equally attracted to both men and women had lower odds of engaging in TS compared to those exclusively attracted to men. Bisexual men may benefit from more diverse sources of emotional and financial support, including female partners, which can reduce reliance on transactional arrangements. Bisexual MSM in sub-Saharan Africa have been observed to engage in fewer high-risk behaviors. A systematic review found that on average, bisexual MSM in sub‐Saharan Africa have lower HIV prevalence and report less receptive anal intercourse than exclusive MSM [28]. These differences in risk profile are broadly consistent with our observation of lower TS among those with bisexual attractions. Conversely, a study in Nigeria found that MSM identifying as bisexual had over twice the odds of transactional sex compared to exclusively gay MSM. This may be due to Nigeria’s Same-Sex Marriage (Prohibition) Act (2014), which criminalizes same-sex relationships with up to 14 years’ imprisonment, and common police mass raids. Many bisexual men, therefore, adopt or retain heterosexual partnerships and use discreet, paid encounters with male partners to satisfy same-sex desire while minimizing public exposure. This dynamic makes TS a safer way to meet male partners under intense surveillance, hence its high prevalence in Nigeria’s bisexual group. Ghana also criminalizes same-sex activity, but enforcement is generally less aggressive, and large-scale raids are rare. MSM social venues (e.g., house parties, community-based organizations) operate with lower risks, allowing gay-identified men to find casual partners without explicit payment.

Our study also observed that participants who reported having multiple sexual partners in the last 6 months were more likely to engage in transactional sex. MSM reporting four or more partners had nearly double the odds of TS compared to those with only one partner. This mirrors findings from other studies of MSM in West Africa. The CohMSM prospective cohort (Burkina Faso, Côte d’Ivoire, Mali, Togo) found that MSM with multiple male partners had significantly higher odds of reporting TS[29]. Similarly, this finding is also consistent with findings from Smith et al. in a cross-sectional study that observed that the larger the sexual network of MSM, the greater the probability of TS with a member who also engages in transactional sex[30]. Generally, having many partners expands opportunity for transactional encounters, both in terms of more encounters, each representing an opportunity for exchange, and reflecting a propensity for casual or group-sex settings that may involve exchanges.

In our study, MSM who had not heard of PrEP were less likely to engage in transactional sex compared to those who were aware of PrEP. This suggests that PrEP awareness may be more prevalent among individuals with higher HIV risk profiles, including those involved in transactional sex, who are more frequently reached by targeted prevention efforts. In Ghana, men who learn about PrEP often are connected to peer educators and prevention programs[18], suggesting that outreach is prioritized toward higher risk MSM. PrEP awareness in this context may serve as a marker of engagement with HIV prevention services among those already facing elevated vulnerability. Importantly, PrEP education and awareness among MSM are still scaling up in Ghana.

PrEP education and awareness among MSM are still scaling up in Ghana. Romo et al. in a study in Kenya found PrEP awareness to be 69% among MSM[31], higher than the 44% reported in Ghana[18]. As PrEP roll-out continues, attention should be paid to ensuring that higher-risk MSM, including sex workers, maintain safe practices.

The strongest protective factor we found was knowledge of HIV testing sites. While this specific association has not been widely reported, it is consistent with the idea that awareness and use of HIV services are linked to safer behaviors. Knowing where to test indicates engagement with the healthcare system and suggests better HIV literacy, which may discourage transactional sex. Conversely, MSM who do not know testing locations might be more marginalized and less informed about prevention, making them more likely to rely on sex-for-gain. This underscores the need for outreach; expanding community-based testing campaigns and publicizing MSM-friendly clinics could have dual benefits of increasing testing uptake and indirectly reducing risky practices like TS.

While TS was not significantly associated with HIV or syphilis in multivariable models, the direction of association was consistent with prior global findings. A systematic review from 28 countries found that MSM engaging in transactional sex had 30% higher odds than those who did not engage in transactional [8]. Research conducted in China and sub-Saharan Africa demonstrates that transactional sex leads to higher rates of HIV and STIs including syphilis, mainly because of economic disadvantage and social discrimination [32, 33]. The absence of significance may be due to confounding protective factors such as age, PrEP awareness, or health service engagement. MSM aged 35 and above had significantly higher odds of HIV infection, suggesting cumulative risk over time. Similarly, PrEP awareness and HIV testing knowledge were protective against both HIV and syphilis. Studies in Kenya and sub-Saharan Africa have shown that improved PrEP literacy and access to tailored services are strongly associated with greater willingness to use PrEP and reduced HIV vulnerability among MSM[34, 35].

## Recommendations

### Scale up PrEP awareness and delivery through MSM-friendly services

*Rationale*. Awareness of PrEP was more common among MSM engaged in transactional sex, suggesting that PrEP education efforts are already reaching those at elevated HIV and STI risk. However, awareness alone does not necessarily translate into access or uptake.

*Recommendation.* Integration of PrEP education and distribution into MSM-friendly clinics, mobile outreach teams, and peer-led initiatives should be prioritized, with particular focus on areas with high transactional sex prevalence such as Greater Accra, to ensure that high-risk MSM are not only aware of PrEP but also able to access and use it effectively.

### Strengthen community-based HIV testing and stigma-free health services

*Rationale*. MSM who did not experience stigma were less likely to engage in transactional sex, underscoring the protective role of stigma-free environments. Knowledge of testing sites was also associated with reduced engagement in transactional sex, highlighting the importance of accessible and supportive services. *Recommendation*. Expand community-based HIV testing campaigns with a focus on anonymity and confidentiality. Train health workers in anti-stigma practices and fund MSM-focused service centers that offer comprehensive sexual health care in safe, respectful environments, recognizing that stigma-free services are protective against engagement in transactional sex.

### Expand access to economic empowerment programs for young MSM

*Rationale.* The study found that transactional sex is most common among younger MSM, particularly those aged 18–24. While income was not directly associated with TS in this study, younger MSM may face limited livelihood options and structural vulnerability that may increase reliance on TS. *Recommendation.* Develop and fund interventions focused on youth vocational training, educational support, and psychosocial services that address the broader structural and social drivers of vulnerability among young MSM, thereby reducing their reliance on transactional sex.

## Limitations and strength

This study has several limitations. First, the sensitive nature of questions related to transactional sex may have introduced social desirability bias, potentially leading participants to underreport such behaviors. To mitigate this, participants were assured of the confidentiality and anonymity of their responses. Second, while respondent-driven sampling (RDS) is effective for reaching hidden populations like MSM, it may introduce recruitment bias stemming from the social networks of initial seeds and unequal chain recruitment. Third, the dataset lacked key structural and psychosocial variables specifically housing instability, employment status, and internalized stigma, which have been shown to influence engagement in transactional sex among MSM. Their absence may limit our ability to fully capture the underlying drivers of transactional sex in this population. Additionally, our main outcome variable was based on a dichotomous measure of transactional sex, which may not capture the full spectrum or frequency of such exchanges. As a secondary analysis of a national survey, the study also lacked qualitative data that could have provided deeper insights into the motivations and contexts underlying engagement in transactional sex. Nonetheless, the study benefits from a large, geographically diverse sample and remains the most current national-level analysis on transactional sex and HIV/STI outcomes among MSM in Ghana.

## Conclusion

This nationally representative study provides critical insights into the structural and behavioral factors driving transactional sex among MSM in Ghana. Transactional sex was most prevalent among younger, less educated, and urban-dwelling MSM, particularly those with multiple sexual partners, limited HIV knowledge, and histories of forced sex. While transactional sex was not significantly associated with HIV or syphilis in adjusted models, it clustered with multiple known risk factors, underscoring its role in shaping vulnerability within this population. These findings highlight the need for targeted, context-sensitive interventions that address both the economic drivers of transactional sex and the broader structural inequalities, particularly among young and marginalized MSM. Expanding access to education, PrEP, HIV testing, and stigma-free healthcare, especially in urban centers like Greater Accra, will be essential to reducing HIV risk and improving health equity. This study strengthens the evidence base for integrating structural interventions with biomedical prevention strategies in Ghana’s national HIV response and contributes to the growing regional literature on HIV vulnerability among sexual minority populations in West Africa.

## Data Availability

The datasets generated and analyzed during the current study are not publicly available due to the high risk of persecution and severe adverse social consequences related to the socio-political sensitivity of the topic of same-sex behaviors in Ghana; however, data are available from the corresponding author on reasonable request.

## Abbreviations

AIDS: Acquired immune deficiency syndrome
ART: Antiretroviral therapy
CDC: Centers for Disease Control and Prevention
CSO: Civil Society Organization
CohMSM: Cohort of men who have sex with men
ERC: Ethics review committee
FHI: Family Health International
FSW: Female sex workers
GHS: Ghana Health Service
HIV: Human Immunodeficiency Virus
IRB: Institutional Review Board
LMIC: Low- and middle-income countries
MSM: Men who have sex with men
MST-TS: Men who have sex with men engaging in transactional sex
NMIMR: Noguchi Memorial Institute for Medical Research
NACP: National AIDS Control Programme
PrEP: Pre-exposure prophylaxis
RDS: Respondent-driven sampling
RDS: Respondent driven sampling
REDCap: Research Electronic Data Capture
SQL: Structured query language
STATA: Statistics and data
STI: Sexually transmitted infections
UNAIDS: Joint United Nations Programme on HIV/AIDS
WHO: World Health Organization
